# Localization of Claudin‐3 and Claudin‐4 within the Small Intestine of newborn piglets

**DOI:** 10.14814/phy2.14717

**Published:** 2021-02-01

**Authors:** Brodie Deluco, Kezia R Fourie, Olena M Simko, Heather L Wilson

**Affiliations:** ^1^ Vaccine and Infectious Disease Organization‐International Vaccine Centre (VIDO‐InterVac) University of Saskatchewan Saskatoon SK Canada

**Keywords:** claudin, colostrum, immunity, piglet, small intestine, tight junction, transport

## Abstract

Piglets must acquire passive immunity through colostrum within hours after birth to survive. How colostral macromolecules traverse the small intestinal epithelium may include nonselective pinocytosis and paracellular transport through tight junction proteins located between epithelial cells. Claudin proteins‐3 and ‐4 contribute to the epithelial tight junctions (TJs) on the apical aspect of lateral surfaces of intestinal epithelial cells (IECs) where they help regulate ion and macromolecule movement across the intestinal epithelium. Throughout the small intestine of newborn piglets, Claudin‐3 was localized to the lateral and basolateral surface of intestinal epithelial cells as well as the membrane of large vacuoles. In the duodenum and jejunum, Claudin‐4 was localized to the apical surface independent of tight junction regions. In the ileum, Claudin‐4 was localized to the lateral and basolateral surfaces indicating region‐specific differences and noncanonical patterns of Claudin‐4 localization independent of tight junction regions. Understanding the timing of changes in surface localization of Claudin‐3 and Claudin‐4 and how they may coincide with changes in small intestinal permeability may help develop new protective strategies against infectious diseases within newborn piglets.

## Graphical abstract

The newborn piglet small intestine epithelial cells do not exclusively express Claudin‐3 and Claudin‐4 at the tight junction region which may lead to leaky gut.

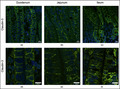

## 1 INTRODUCTION

Epithelial tight junction (TJ) proteins are composed of strands located on the apical aspect of lateral surfaces of intestinal epithelial cells (IECs) where they interact with other strands on neighboring IECs to regulate ion and macromolecule movement across the intestinal epithelium (Farquhar & Palade, 1963; Garcia‐Hernandez et al., 2017; Staehelin, 1973). The composition and organization of these strands is what determines the closure of TJ proteins (Farquhar & Palade, 1963; Garcia‐Hernandez et al., 2017; Staehelin, 1973). The formation of TJ proteins is very dynamic and often changes throughout the life cycle of the intestinal epithelium as well as in response to enteropathogenic infections thus influencing intestinal epithelial homeostasis and barrier properties (Turner et al., 2014; Wang et al., 2016). In the small intestine, the apical absorbing villus epithelial cell pores allow passive transport of small molecules such as monosaccharides (<6 Angstroms), whereas pores in the crypts, which are inaccessible to luminal content in the healthy gut, allow passage of larger macromolecules (50–60 Angstroms) (Fihn et al., 2000). Epithelial TJ proteins, such as claudin proteins, are highly expressed within the respiratory, gastrointestinal (GI), and uterine tract epithelium. The human claudin family includes 26 transmembrane proteins with many of these proteins also found in pigs (Farquhar & Palade, 1963; Garcia‐Hernandez et al., 2017; Staehelin, 1973). Claudins primarily regulate the TJ high‐capacity pore pathway that allows small, uncharged solutes and specific ions to pass between IECs whereas zonodulin‐1 and occludin regulate the TJ low‐capacity leak pathway that is permeable to larger macromolecules (Furuse et al., 1994; Garcia‐Hernandez et al., 2017; Shen et al., 2011). Claudins‐1, ‐3, ‐4, ‐5, and ‐18 are categorized as tightening claudins which function by tightening the paracellular cleft of IECs via changes in the distribution and arrangement of charged amino acids thus preventing pore formation (Anderson & Van Itallie, 2009). Claudin‐3 is primarily expressed in the distal parts of the GI tract including the colon, sigmoid, and rectum, and it is also expressed at lower levels within the small intestine, whereas Claudin‐4 is primarily expressed within the small intestine (Lu et al., 2013; Markov et al., 2010). Claudin‐3 gene expression has found to increase in jejunum of piglets from birth to 21 days of life with no differences in gene expression being observed in the ileum during this period (Turner et al., 2014; Wang et al., 2016). Claudins‐3 and ‐4 are located with zonodulin‐1 at tight junctions and they contribute to barrier function, however, they are also distributed along the lateral cell membranes of IECs (Van Itallie et al., 2019; Milatz et al., 2010). Whether Claudin‐3 and ‐4 non‐canonical surface localization contribute to macromolecule movement across the newborn piglet gut wall should be explored.

The newborn piglet small intestine is semi‐permeable to allow absorption of colostral macromolecules provided by the sow (Nguyen et al., 2007; Sangild, 2003). This semi‐permeability or “leakiness” within the small intestine has been reported to last up to 36 h after birth before small intestinal closure occurs; although the timing of this may have reduced with the rearing of increasingly precocious piglets (Lecce et al., 1964; Lecce & Morgan, 1962). We previously showed that Claudin‐4 was largely localized to the apical aspect of jejunal IECs for the first 2 days of life after which it became localized to the lateral region of IECs (Pasternak et al., 2015). In the ileum, Claudin‐4 was located on the lateral surface of IECs as early as 24 h of age but it was absent from the apical surface of IECs (Pasternak et al., 2015). Herein, we intend to establish whether there are region‐specific differences in the localization of Claudin‐3 and Claudin‐4 within the small intestine of the piglet within 7 h after birth using confocal microscopy. This information will further allow us to investigate whether Claudin‐3 and ‐4 noncanonical localization contributes to intestinal permeability in the newborn piglet.

## 2 MATERIALS AND METHODS

### 2.1 Tissue collection

This work was approved by the University of Saskatchewan's Animal Research Ethics Board and adhered to the Canadian Council on Animal Care Guidelines for humane animal use. Piglets (*n* = 6) were humanely killed by a nonpenetrating Zephyr machine (i.e., captive bolt) coupled with exsanguination at 7‐h after birth. The small intestine was excised and 10 cm in length was removed from the duodenum (10 cm distal from the pyloric sphincter), the ileum (10 cm proximal from the ileocecal fold) were obtained from 7‐h old nursing piglets (Kararli 1995). The 10 cm segment removed from the jejunum was selected by taking the 50% of the length of the entire small intestine as we have previously used for newborn piglet intestinal analysis (Pasternak et al., 2016). The tissues were placed within separate Erlenmeyer flasks with DMEM media. Small cross‐sections (1 cm^2^) of the intestinal segments from each piglet were placed within Tissue‐Loc biopsy cassettes (ThermoFisher Scientific 58931) for immunohistochemistry (IHC) purposes. Tissues were fixed in 10% buffered formalin (Sigma‐Aldrich) for 48 h, and then, dehydrated in a series of increasing concentrations of alcohol (1× 70% Ethanol, 1× 80% Ethanol, 2× 95% Ethanol, 3× 100% Ethanol, 1× Ethanol ‐Xylene, 2× Xylene, and 4× Paraplast® (Sigma‐Aldrich)) before embedding in paraffin.

### 2.2 Immunohistochemistry

Small intestinal tissue sections of 7‐h old piglets (*n* = 6) were deparaffinized in decreasing concentrations of alcohol (4× xylene (5 min per wash), 3× 100% Ethanol (1 min per wash), 2× 95% Ethanol (1 min per wash), and 1× 70% Ethanol (5 min per wash)). Superfrost Plus microscope slides (ThermoFisher Scientific 22‐034‐979) were blocked for 3 h at room temperature in 5% (w/v) blotting grade blocker nonfat dry milk (BIO‐RAD 170‐6404) in 1× PBS, pH 7.4). Next, heat‐induced antigen‐retrieval was performed in Tris‐EDTA buffer (10 mM Tris, 1 mM EDTA Solution, 0.05% Tween 20, pH 9.0; Sigma) for 13 min at power level 6 within a Panasonic microwave. The following primary antibodies with DAKO antibody diluent (Agilent S302283‐2) were added to the slides: rabbit anti‐Claudin‐4 antibody (ab53156) (1:250 dilution), rabbit anti‐Claudin‐3 antibody (ab15102) (1:100 dilution), and rabbit anti‐Villin antibody (ab233155) (1:100 dilution). Slides were then incubated overnight at 4°C. The following day, slides were washed 3× for 5 min in 1× PBSA and then incubated in 1:500 dilution of Alexa 555‐labeled goat anti‐rabbit immunoglobulin G (IgG) (4030‐02; Southern Biosystems) with DAKO antibody diluent at 4°C for 4 h. Slides were washed 3× for 5 min in 1× PBSA, and then, dehydrated for 1 min in increasing concentrations of alcohol (3× 95% Ethanol (10 s per wash), 3× 100% Ethanol (10 s per wash), and 4× Xylene (10 s per wash)) before coverslips (20 × 53 mm) were added with VECTASHIELD® Vibrance™ Antifade Mounting Media with 4′,6‐diamidino‐2‐phenylindole (DAPI; H‐1800; Vector Laboratories). Intestinal sections were imaged using a Leica confocal microscope with blue color corresponding to the DAPI signal.

## 3 RESULTS

To determine whether there are region‐specific differences in the localization of Claudin‐3 and Claudin‐4 within the small intestine of 7‐h old piglets, paraffin‐embedded intestinal slices were probed with Claudin‐3 and Claudin‐4‐specific antibodies. Intestinal tissues incubated with Alexa 555‐labeled goat anti‐rabbit IgG without a rabbit primary antibody shows a weak fluorescent signal within the *lamina propria* regions of the duodenum, jejunum, and ileum which may suggest nonspecific binding of the secondary antibody to connective tissues or cells or background fluorescence at 555 nm (data not shown).

**FIGURE 1:**
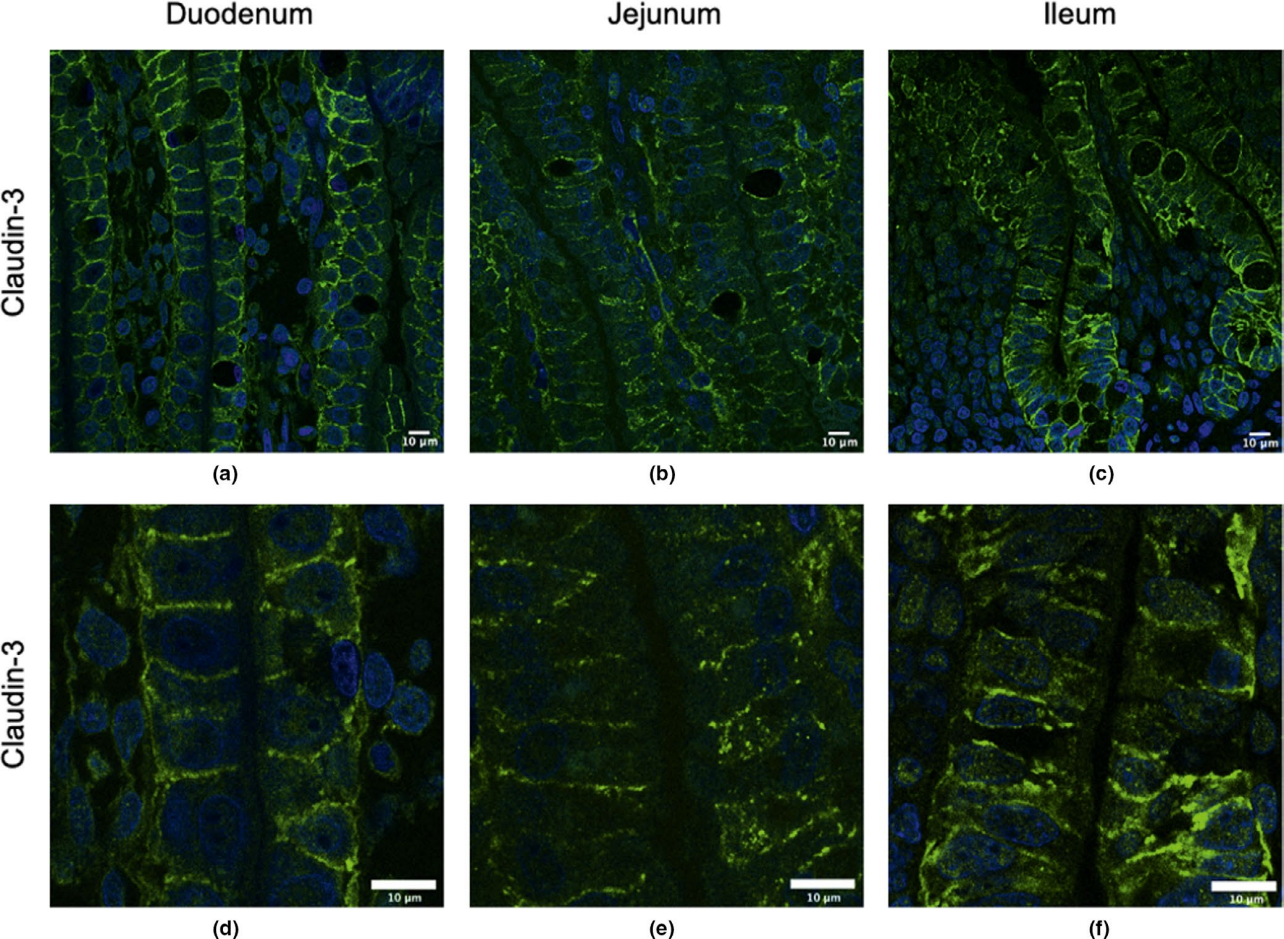
Claudin‐3 surface localization in the duodenum, jejunum and ileum of 7‐h old piglets. These images are representatives of IHC‐p performed on intestinal tissue samples from six different biological replicates. The primary antibody is rabbit anti‐Claudin‐3 and the secondary antibody is Alexa‐555 goat anti‐rabbit (green). Images from duodenum (a, d), jejunum (b, e), and ileum (c, f) are shown. Nuclear stain: DAPI (blue). All images were taken with a Leica Confocal Microscope at 63×.

**FIGURE 2:**
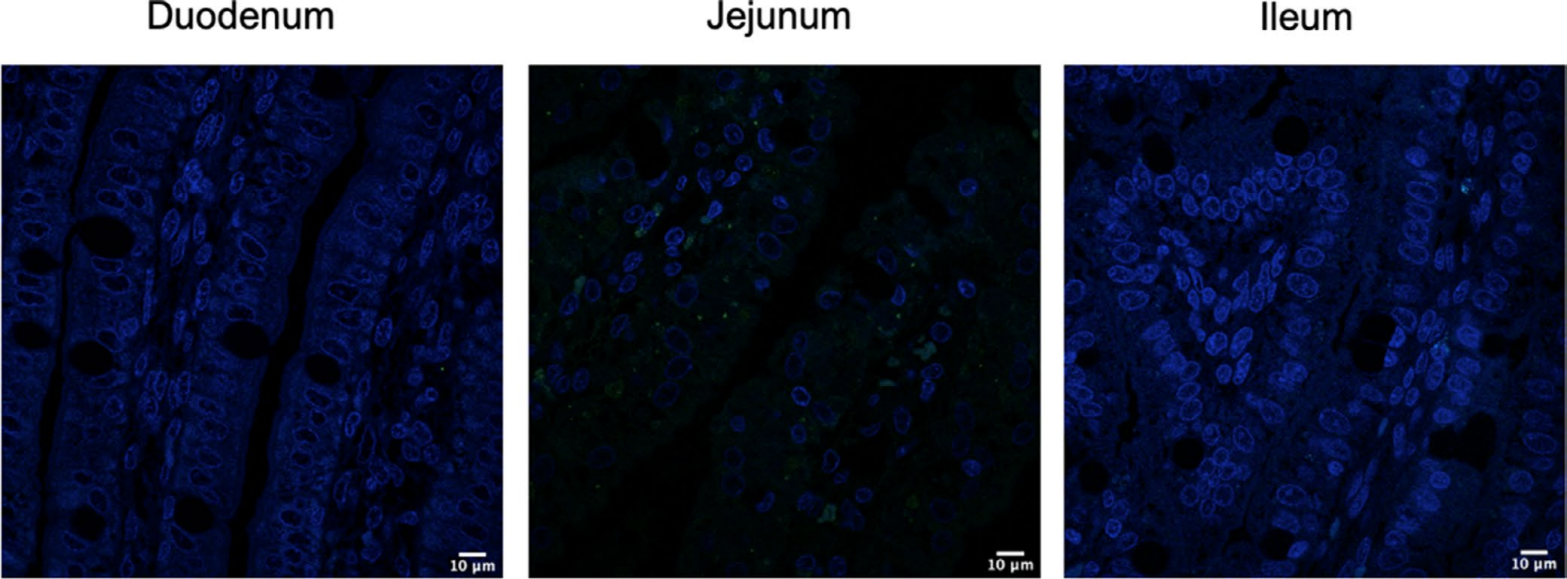
Control immunohistochemistry for duodenum, jejunum, and ileum of 7‐h old piglets. These images are representatives of IHC‐p performed on intestinal tissue samples from three different biological replicates. The primary antibody, rabbit anti‐Claudin‐3 and rabbit anti‐Claudin‐4 are absent but the secondary antibody Alexa‐555 goat anti‐rabbit is present (green). Images from duodenum, jejunum, and ileum are shown. Nuclear stain: DAPI (blue). All images were taken with a Leica Confocal Microscope at 63×.

Strong lateral and basolateral localization of Claudin‐3 was observed in the duodenum (Figure 1a), jejunum (Figure 1b), and the ileum (Figure 1c), but it did not appear to be apically localized in any regions of the small intestine. Claudin‐3 was also located around the outer membrane of vacuoles in all areas of the small intestine. These regions are more clearly visible in the zoomed in regions in Figure 1d–f. Figure 2a–c show the control duodenum, jejunum, and ileum, respectively, where the secondary antibody is present. Very little background fluorescence is observed in any tissue.

**FIGURE 3:**
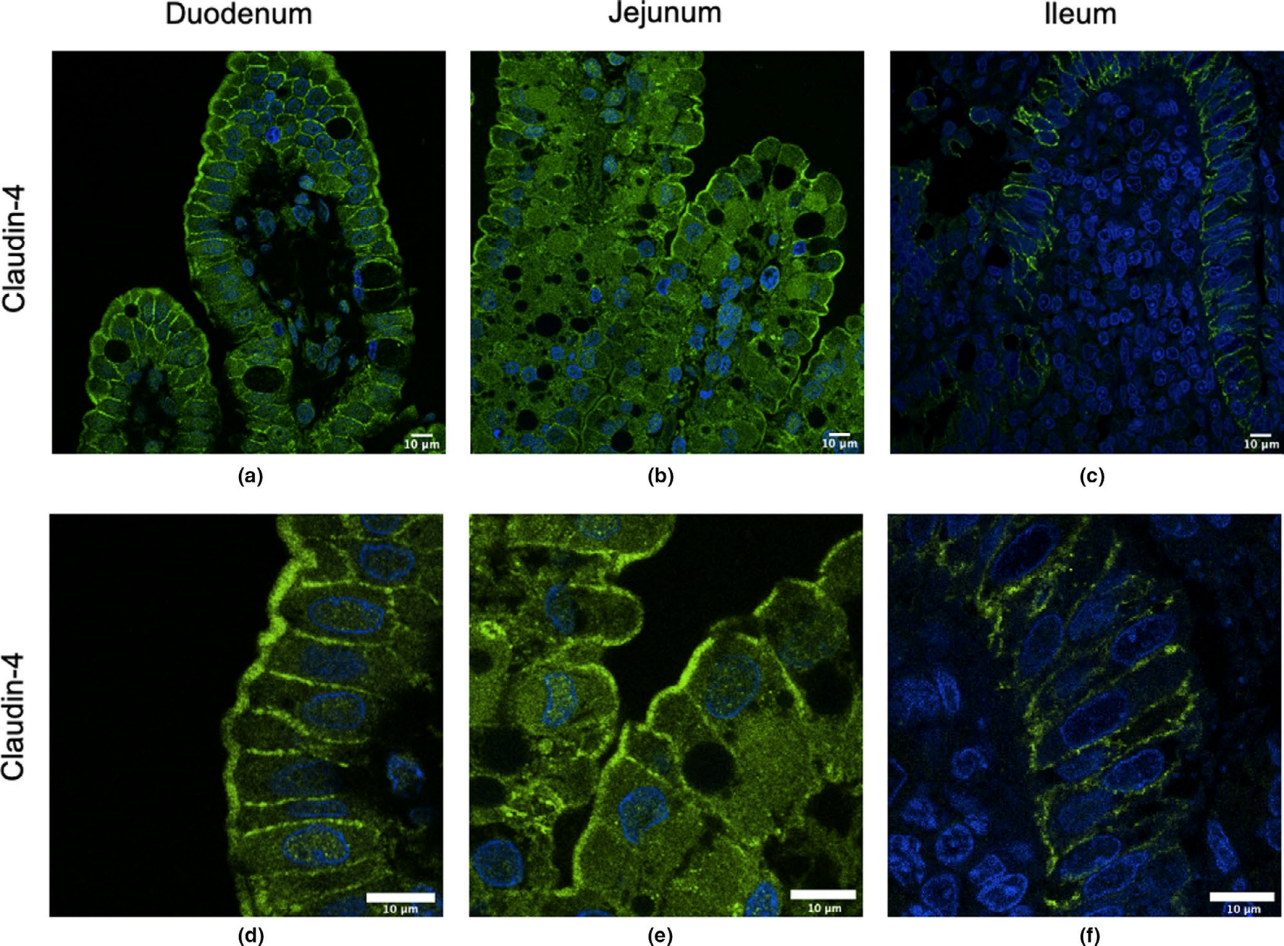
Claudin‐4 surface localization in the duodenum, jejunum, and ileum of 7‐h old piglets. These images are representatives of IHC‐p performed on intestinal tissue samples from six different biological replicates. The primary antibody is rabbit anti‐Claudin‐4 and the secondary antibody is Alexa‐555 goat anti‐rabbit (green). Images from duodenum (a, d), jejunum (b, e), and ileum (c, f) are shown. Nuclear stain: DAPI (blue). All images were taken with a Leica Confocal Microscope at 63×.

In the duodenum (Figure 3a) and jejunum (Figure 3b) of 7‐h old piglets, Claudin‐4 was located laterally between IECs as well as strongly present at the apical surface of IECs (villi tips). In the ileum (Figure 3c), Claudin‐4 was present in the lateral regions as well as at the basolateral surface of IECs but not at the apical surface. These regions are more clearly visible in the zoomed in regions in Figure 3d–f.

## 4 DISCUSSION

The porcine small intestine is lined with a single layer of columnar epithelial cells that forms a dynamic barrier allowing for selective absorption of nutrients, while normally restricting permeability to most macromolecules including antigens and pathogens. Fetal piglets do not share circulation with the sow and thus their serum is devoid of maternal antibodies and many serum proteins at birth. As a result, newborn piglets must ingest colostrum to obtain IgGs as well as other macromolecules such as albumin, cytokines, and antimicrobial peptides all of which are essential for protection against disease (Nechvatalova et al., 2011). The newborn piglet gut has evolved to be semi‐permeable immediately after birth to accommodate this need for the rapid transfer of colostral macromolecules. When the gut is no longer able to take up or internalize macromolecules (referred to as gut‐closure), has been reported to take place approximately 2 days after birth with the proximal intestine undergoing gut closure more rapidly than the distal small intestine (Lecce, 1973; Lecce et al., 1964).

Studies in many animal species typically show that Claudin‐3 and ‐4 are localized to the lateral regions of IECs. For example, studies in mice showed that Claudin‐3 is localized predominantly at the intercellular junctions of the colonic and ileal epithelia (Shukla et al., 2016). In rat jejunal IECs, Claudin‐3 and ‐4 are largely located in the lateral regions and Claudin‐3 is also located at the basolateral surface of IECs (Markov et al., 2016). In dogs, Claudin‐3 is localized at the apical lateral region of duodenal IECs and in the basolateral region of the paracellular surface in the colon (Ohta et al., 2011). In chicks, Claudin‐3 was found to be located in the lateral regions after hatching (Ozden et al., 2010). The piglet small intestinal epithelium showed very low expression of Claudin‐3, but it appears to be laterally localized. We previously reported that Claudin‐4 localized to the lateral and apical surfaces in the jejunum but only to the lateral surface in 24–48‐h old piglets (Pasternak et al., 2015). This current research confirms what was observed in the 1‐day old piglet jejunal epithelia and it was expanded to include the duodenum where we observed apical as well as lateral localization. However, we observed lateral and basolateral localization of Claudin‐4 in the ileum, the latter of which was not reported. This current report is also the first to report that Claudin‐3 is localized at the lateral and basolateral surface throughout the small intestine as well as around the outer membrane of vacuoles in newborn piglets. It is not clear what role these claudins may play while localized outside of the TJ regions but it is likely independent of barrier function (Weber, 2012). Claudin proteins along the lateral surface and/or apical or basolateral surfaces may act as a reserve to re‐enforce TJ regions once gut‐closure is initiated, or they may play a role in signaling events that control epithelial homeostasis (Weber, 2012).

Claudin protein expression within the small intestine plays a vital role in controlling barrier function and mucosal homeostasis. Spatial claudin expression is not well elucidated in the pig but we predict that the precise organization of claudin proteins may be different in the newborn piglet when the small intestine is semi‐permeable to allow for the uptake of colostral macromolecules and maternal cells. Identifying how spatial control of claudin proteins in the small intestine is regulated is important for understanding the transition from the semi‐permeable gut prior to gut‐closure. Whether Claudin‐3 and Claudin‐4 apical and basolateral localization directly impacts small intestinal permeability deserved further analysis as this understanding of small intestinal permeability may help develop new protective strategies against infectious diseases within newborn piglets.

## COMPETING INTERESTS

The authors declare that they have no competing interests.

## AUTHOR CONTRIBUTIONS

BD and HLW conceived of and designed the experiments. BD carried out all assays with assistance from KRF and OMS. BD analyzed all data and drafted the manuscript. All authors read and approved the final manuscript.

## ETHICAL APPROVAL

This work was approved by the University of Saskatchewan's Animal Research Ethics Board and adhered to the Canadian Council on Animal Care Guidelines for humane animal use.

## CONSENT FOR PUBLICATION

All authors have given consent for publication.

## Data Availability

The data sets used and/or analysed during the current study are available from the corresponding author on reasonable request.
